# Iron Overload-induced Ferroptosis as a Target for Protection against Obliterative Bronchiolitis after Orthotopic Tracheal Transplantation in Mice

**DOI:** 10.2174/0115665240304363240524103203

**Published:** 2024-06-03

**Authors:** Yun You, Guoliang Wang, Qi Cui, Xiangfu Sun, Li Wan, Quanchao Sun

**Affiliations:** 1 Department of Vascular Surgery, Union Hospital, Tongji Medical College, Huazhong University of Science and Technology, Wuhan, China;; 2 Center for Liver Transplantation, Union Hospital, Tongji Medical College, Huazhong University of Science and Technology, Wuhan, China;; 3 Tongji Medical College, Huazhong University of Science and Technology, Wuhan, China;; 4 Department of Thoracic Surgery, Union Hospital, Tongji Medical College, Huazhong University of Science and Technology, Wuhan, China

**Keywords:** Iron overload, ferroptosis, obliterative bronchiolitis, lung transplantation, CLAD, tracheal transplantation

## Abstract

**Introduction:**

The major complication of Obliterative Bronchiolitis (OB) is characterized by epithelial cell loss, fibrosis, and luminal occlusion of the terminal small airways, which limits the long-term survival of the recipient after lung transplantation. However, the underlying mechanisms are still not fully clarified. This research aims to investigate whether iron overload-induced ferroptosis is involved in OB development and provide a new target for OB prevention.

**Materials and Methods:**

Allograft orthotopic tracheal transplantation in mice was applied in our study. Ferrostatin-1 and deferoxamine were administrated to inhibit ferroptosis and get rid of ferric iron, while iron dextran was used to induce an iron overload condition in the recipient. The histological examination, luminal occlusion rate, collagen deposition, iron level, ferroptosis marker (GPX4, PTGS2), and mitochondrial morphological changes of the graft were evaluated in mice.

**Results:**

Our research indicated that ferroptosis and iron overload contribute to OB development, while ferroptosis inhibition and iron chelator could reverse the changes. Iron overload exacerbated OB development after orthotopic tracheal transplantation *via* promoting ferroptosis.

**Conclusion:**

Overall, this research demonstrated that iron overload-induced ferroptosis is involved in OB, which may be a potential therapeutic target for OB after lung transplantation.

## 1 INTRODUCTION

Lung transplantation is the only established lifesaving strategy for variable pulmonary diseases at the end-stage. The long-term survival rate and prognosis of lung transplantation are restricted by the progress of Chronic Lung Allograft Dysfunction (CLAD) [1-3]. Obliterative Bronchiolitis (OB), characterized by terminal bronchioles fibrosis, is the main reason for the death of CLAD recipients after lung transplantation. The pathological mechanism of OB development is pretty complicated, including alloimmunity, inflammation, proliferation, and fibrosis [2, 4, 5]. The complex pathogenesis of OB is still unclear, and there has not been anyimprovement in OB therapy [6]. Thus, it is critical to develop a new target to prevent OB for successful lung transplantation.

Iron is an indispensable metallaic nutrition element in most biochemical reactions, and its main biological function is to transport oxygen. In addition, iron overload can promote Reactive Oxygen Species (ROS) generation *via* the Fenton reaction and further wreak havoc on cells [7-9]. Ferroptosis was a novel iron-dependent programmed cell death mode characterized by abnormal iron deposition, excessive lipid peroxides, and ROS generation, which is involved in many pathological processes, such as tumorigenesis, ischemia-reperfusion injury, fibrosis, and neurodegeneration diseases [10-13]. Distinguished from other kinds of cell death, condensed mitochondrial membranes, reduced mitochondrial crista volume, and shrunken mitochondria are the main morphological features of ferroptosis. Iron accumulation, increased ROS production, and over-lipid peroxidation play a critical role in initiating ferroptosis [14-16]. Furthermore, down-regulated expression of the lipid peroxidation scavenger Glutathione Peroxidase 4 (GPX4) and increased Prostaglandin-endoperoxide Synthase 2 (PTGS2) levels are defined as key markers of ferroptosis [17-19]. Ferroptosis is initiated when GPX4 expression is suppressed due to a decrease of Glutathione (GSH) level. GPX4 inhibition contributes to excessive lipid peroxidation, leading to cellular membrane damage and even cell death [20, 21]. Moreover, ferrostatin-1 (Fer-1) is reported to be a ferroptosis inhibitor, and the iron chelator Deferoxamine (DFO) is proven to alleviate ferroptosis [22, 23]. Ferroptosis has been shown an impressive effect on the development of numerous pathological processes, including stroke, organ injury, neurodegeneration, and cáncer [24-26]. An increasing number of research studies indicate that targeting iron metabolism is a promising approach to treating many diseases [27, 28]. However, whether ferroptosis is involved in OB after lung transplantation remains unclear.

In our present study, the effect of iron overload and ferroptosis in OB was investigated. According to existing research, this is the first study that has shown that ferroptosis is involved in OB and inhibition of ferroptosis ameliorates OB after orthotopic tracheal transplantation. Furthermore, we found that OB was alleviated by iron chelator DFO but aggravated due to extra iron dextran administration-induced iron overload. Our research also demonstrated that iron overload contributes to OB development through promoting ferroptosis after orthotopic tracheal transplantation. The present study indicates that iron overload-induced ferroptosis could be a novel therapeutic strategy for OB after lung transplantation.

## 2 MATERIALS AND METHODS

### 2.1 Animals

8–10 weeks old and 22–26 g male, specific pathogen-free C57BL/6 and BALB/C mice were obtained from Shulaibao Co., Ltd (Wuhan, China). A simple randomization method was applied, and all of the animals were kept in a temperature-controlled (22±2ºC) and humidity-controlled room facility (Tongji Medical College, Huazhong University of Science and Technology, Wuhan, People’s Republic of China), and fed with fresh standard laboratory food and water. An animal model was established in accordance with the Guideline for Use and Care of Laboratory Animals (NIH publication, eighth edition), and was approved by the Institutional Animal Care and Use Committee at Tongji Medical College [20^23^] IACUC Number: 3689. The sample size of experimental animals: 8–10 weeks old and 22-26 g male specific pathogen-free C57BL/6 and BALB/C mice were obtained from Shulaibao Co., Ltd (Wuhan, China).

### 2.2 Orthotopic Tracheal Transplantation Model and Groups

The surgical procedure of orthotopic tracheal transplantation was previously reported by our lab. Briefly, the donor mice were euthanized with pentobarbital sodium (80 mg/kg, i.p.) and fixed under a microscope. Six cartilage rings were harvested *via* cervical midline incision. Then, the grafts were flushed, dried, and preserved on ice covered with wet gauze. Later, the recipients were also anesthetized with pentobarbital sodium (50 mg/kg, i.p.) to reach analgesia with spontaneous breath. A cervical midline incision was performed to expose the trachea, which was cut from the second cartilage ring below the cricoid cartilage. Lastly, the tracheal was transplanted end-to-end with 11–0 prolene suture in the recipients [29, 30].

The recipients were designed into different groups: syngraft group, C57BL/6 to C57BL/6; allograft group, BALB/C to C57BL/6; Fer-1+syngraft/allograft group; DFO+syngraft/allograft group; FeDex+syngraft/allograft group; FeDex+Fer-1+allograft group. Five recipients in a cage for each group, and the total number of mice used in our experiment was one hundred, including donors and recipients. Two recipients in the FeDex+Fer-1+allograft group and three recipients in the FeDex+allograft group dead post transplantation, which were excluded. Recipients in the DFO+syngraft/allograft group were assigned deferoxamine (Sigma-Aldrich D9533, 50 mg/kg·2 day^−1^, i.p.) from day 0 post trachea transplantation, while the control groups were treated with saline [31, 32]. In the Fer-1+syngraft/allograft group, mice were intraperitoneally injected with Ferrostatin-1 (Sigma-Aldrich SML0583, 5 mg/kg·day^−1^) from day 0 to day 28 after trachea transplantation [33, 34]. Recipients in the iron overload group (FeDex+syngraft/allograft group) were administered with iron dextran (Merck D8517, 100 g/kg·3 day^−1^, i.p.) from day 0 post trachea transplantation [35, 36]. In the FeDex+Fer-1+allograft group, mice were treated with both iron dextran and Fer-1 according to the method mentioned above individually. After all, mice were euthanized with a lethal dosage of pentobarbital sodium, and the implants were collected to perform histological and biochemical studies at different time points after trachea transplantation. Yun You and Qi Cui were responsible for the animal experiment, sample, and data collection.

### 2.3 Histology, Masson, and Immunohistochemistry Staining

The harvest tracheal grafts were fixed in 4% paraformaldehyde overnight at 4ºC, embedded, and cut into 4-μm-thick paraffin sections. The slices were then stained with Haematoxylin and Eosin (H&E), and Masson's trichrome staining for morphological and collagen deposition examination. The luminal occlusion rate was measured and calculated as previously reported. Briefly, the percentage of luminal occlusion was defined as follows: [(Area within the cartilage –Area of the residual tracheal lumen) / Area within the cartilage]×100% [37]. All of the histological data was analyzed using Fiji software.

For immunohistochemistry labeling, 4-μm-thick slices were dewaxed with xylene and rehydrated with 100%, 95%, 80%, and 75% ethanol. The microwave method was applied to achieve antigen retrieval, and endogenous peroxidase was inactivated in 3% H_2_O_2_. The non-specific staining was then blocked with 5% bovine serum albumin for 30 minutes at room temperature. Slices were labeled with primary antibodies against GPX4 (Abcam ab125066, 1:500) and PTGS2 (Invitrogen #35-8200, 1:500) at 4ºC overnight. After washing, the samples were then labeled with secondary biotinylated antibody for 30 minutes at 37ºC and stained with 3,30-diaminobenzidine tetrahydrochloride at room temperature for 25s. After all, slices were counter-labeled with haematoxylin at room temperature for 5 minutes and imaged using a Leica SDPTOP HS6 scanner.

### 2.4 Prussian Blue Staining

Tissue slices were incubated with freshly prepared 5% potassium hexacyanoferrate trihydrate and 5% hydrochloric acid for 60 minutes at 37°C. Then, they were flushed with distilled water for 5 minutes until the running water was colorless, and further labeled with a nuclear fast red solution for 2 minutes. After all, sections were dehydrated and covered to detect the iron ion using the Leica SDPTOP HS6 microscope.

### 2.5 Detection of Iron Ion Content, MDA, SOD, and GSH Levels

The relative iron ion concentrations in the trachea were evaluated by an iron assay kit (Jiancheng Bioengineering Institute, A039-2-1) according to the instruction. Lipid peroxidation Malondialdehyde (MDA), Superoxide Dismutase (SOD), and total Glutathione (GSH) levels were tested with an MDA assay kit, SOD assay kit and GSH Assay Kit (Beyotime Institute of Biotechnology). The absorbance was applied to measure MDA (523 nm), SOD (450 nm), and GSH (405 nm) levels by a microplate reader. The MDA, SOD, and GSH levels were normalized by total protein concentrations, which is evaluated with Bicinchoninic Acid (BCA) protein assay kit (ThermoFisher Scientific).

### 2.6 Transmission Electron Microscopy

Tracheal samples were fixed in 0.1 M cacodylate buffer with 2.5% glutaraldehyde and 2% paraformaldehyde. Ultrathin sections were achieved using a Leica Ultracut microtome and incubated with 2% saturated uranyl acetate alcohol and 2.6% lead citrate. After that, the ultrastructure morphology of mitochondria was imaged using a Transmission Electron Microscope (JEOL1010).

### 2.7 Statistical Analysis

All data were analyzed using SPSS software (version 22.0; IBM Corp.) and expressed as the mean ± Standard Deviation (SD). Statistical comparisons among groups were analyzed using a one-way Analysis of Variance (ANOVA) followed by Bonferroni's multiple comparison test. Statistical significance was set at *P<0.05*.

## 3 RESULTS

### 3.1 Ferroptosis Aggravated in OB After Orthotopic Tracheal Transplantation

**Fig. (1):**
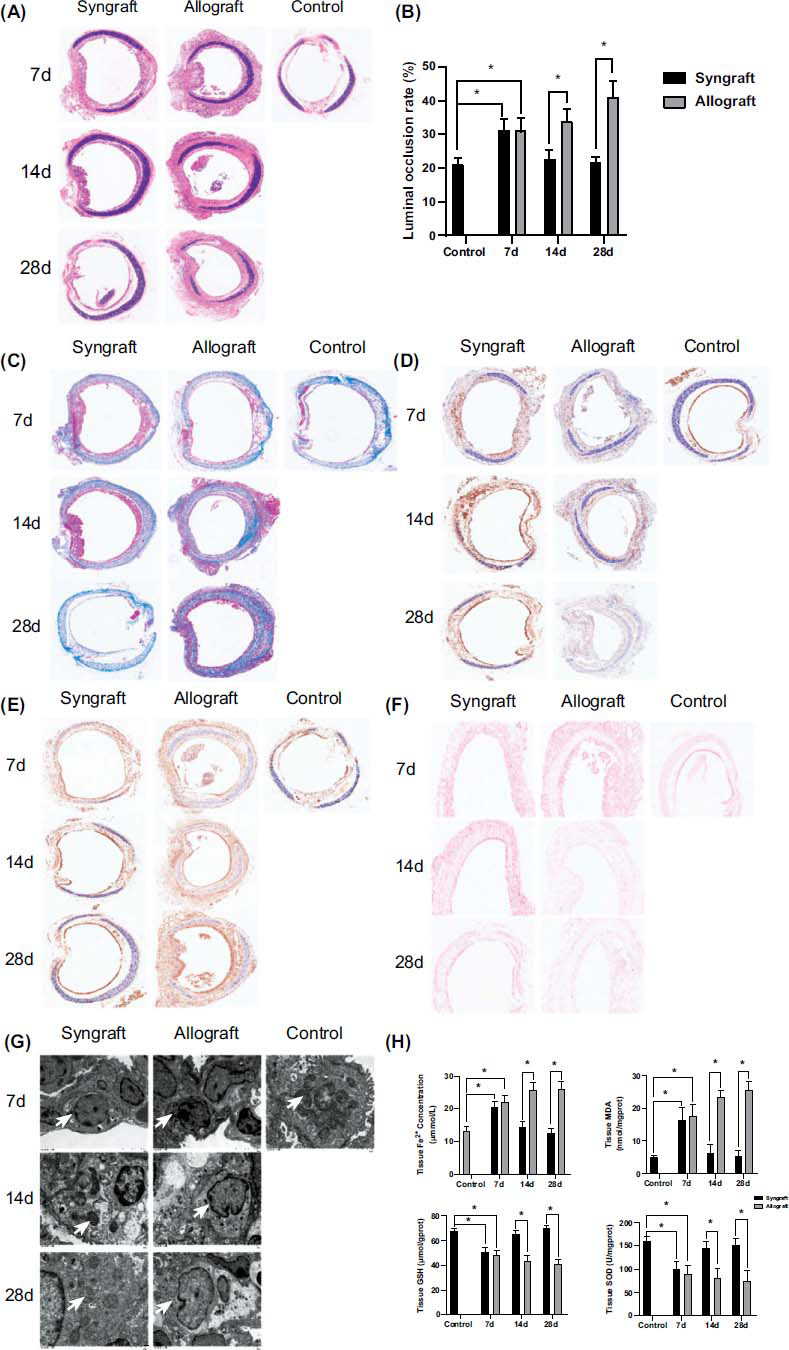
Role of ferroptosis in OB after orthotopic tracheal transplantation. Graft tracheal samples were obtained from mice on days 7, 14 and 28 posttransplant: (**A** and **B**) The luminal occlusion rate was assessed in the HE stained trachea slice. (**C**) Collagen deposition within the tracheal epithelium was evaluated by Masson’s trichrome staining. (**D** and **E**) GPX4 and PTGS2 expression was assessed by immunostaining. (**F**) Quantification of iron deposition in the graft trachea, Prussian blue staining. (**G**) TEM images of trachea tissue. The typical mitochondrial ultra structure was smaller mitochondria volume, condensed membrane, and reduced number of cristae in ferroptosis. ×6000 magnification, scale bar, 1 μm. White arrow, mitochondria. (**H**) Tissue iron concentration, MDA, GSH, and SOD levels were assessed. Data are expressed as mean± SEM. *P <0.05.

We first examined luminal occlusion and ferroptotic morphological and biochemical changes in OB after orthotopic tracheal transplantation. The occlusion rate of the lumen was increased on day 7 posttransplant in the syngraft group, and it was mostly made up of acute inflammation cells and pseudostratified epithelium proliferation, which is different from the control group. However, the structure of the lumen recovered to normal on days 14 and 28 posttransplant in the syngraft group, which was almost the same as the control group. The graft lumen in the allograft group was more occlusive as compared with the control group on day 7 posttransplant. The subepithelial layer was progressively thickening, and the luminal occlusion rate was significantly enhanced in the allograft groupthan the syngraft group on days 14 and 28 posttransplant (Fig. **1A** and **B**). By Masson’s trichrome staining in Fig. (**1C**), the results demonstrated that the tracheal epithelium fibrosis was significantly accelerated in the allograft group than the syngraft group on both days 14 and 28 posttransplant.

GPX4 and PTGS2 are well-known features of ferroptosis. As shown in Fig. (**1D**), the GPX4 expression was inhibited in the syngraft and allograft group compared to those in the control group on day 7 posttransplant. The GPX4 level was recovered on day 14, and there was not any significant difference between days 14 and 28 in the syngraft group. However, the GPX4 level in the allograft group was obviously lower than the syngraft group on days 14 and 28 posttransplant. Immunohistochemical analysis also indicated that the PTGS2 expression level increased in the syngraft group on day 7 than in the control group and recovered to normal levels on days 14 and 28. Meanwhile, the PTGS2 level gradually increased in the allograft group on days 14 and 28, which is much higher compared with the control and syngraft group (Fig. **1E**).

Perls’ Prussian Blue staining showed that iron ion was significantly increased from day 7 to 28 after orthotopic tracheal transplantation in the allograft group than the syngraft group. Even though the iron ion also accumulated on day 7, it can rarely be detected on day 14 or 28 in the syngraft group in Fig. (**1F**). Mitochondrial ultrastructural changes are a typical ferroptosis characteristic. Condensed mitochondrial bilayer densities, generally smaller mitochondria, and fewer mitochondrial cristae were observed in the allograft trachea on days 14 and 28 as compared with the control and syngraft group under transmission electron microscopy (Fig. **1G**). These mitochondria changes could also be detected on day 7 in the syngraft group but turned to be normal on days 14 and 28.

In the syngraft group, the SOD and GSH levels decreased more obviously than the control group on day 7. However, these levels were restored to normal on days 14 and 28 posttransplant. In addition, the iron and MDA content werehigher, but the GSH and SOD content were lower in the allograft group as compared with the syngraft group on days 14 and 28 post orthotopic tracheal transplantation (Fig. **1H**). Overall, the above results indicate that ferroptosis contributes to the OB progression after orthotopic tracheal transplantation.

### 3.2 Inhibition of Ferroptosis Alleviated OB After Orthotopic Tracheal Transplantation

**Fig. (2):**
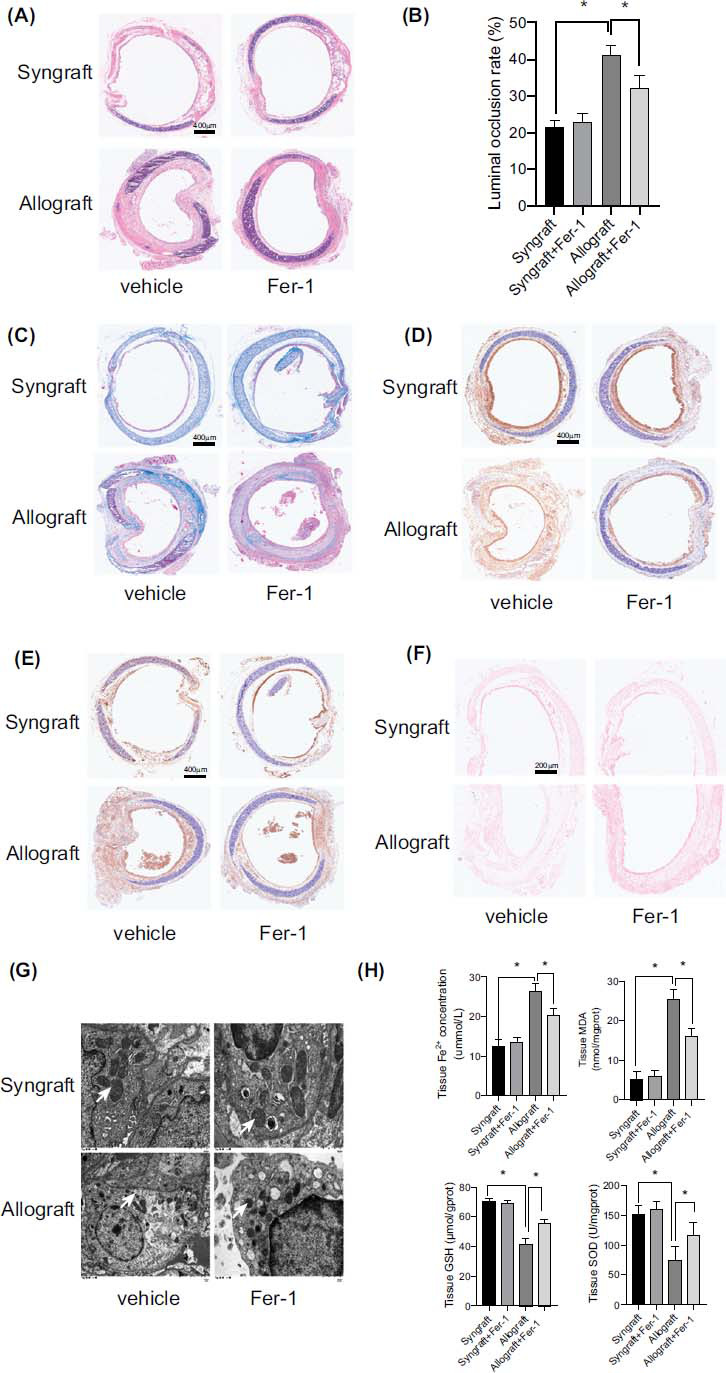
Inhibiting ferroptosis with Fer-1 alleviated OB after orthotopic tracheal transplantation. Graft tracheal samples were obtained from mice 28 days posttransplant: (**A** and **B**) The luminal occlusion rate was assessed in the HE-stained trachea slices. (**C**) Collagen deposition within the tracheal epithelium was evaluated by Masson’s trichrome staining. (**D** and **E**) GPX4 and PTGS2 expression was assessed by immunostaining. (**F**) Quantification of iron deposition in the graft trachea, Prussian blue staining. (**G**) The typical mitochondrial ultra structure was smaller mitochondria volume, condensed membrane, and reduced number of cristae in ferroptosis. ×6000 magnification, scale bar, 1 μm. White arrow, mitochondria. (**H**) Tissue iron concentration, MDA, GSH and SOD levels were assessed. Data are expressed as mean± SEM. *P <0.05.

Next, we determined to assess the role of ferroptosis inhibitors on OB development after orthotopic tracheal transplantation. 28 days was chosen as the observation time point for the analysis of protective effects against OB. Histological analyses demonstrated that the luminal occlusion rates were increased in the allograft group than in the syngraft group. However, the Fer-1 treated allograft group had significantly lower luminal occlusion rates than the allograft group (Fig. **2A** and **B**). By Masson’s trichrome staining in Fig. (**2C**), the results indicated that the tracheal epithelium fibrosis was significantly aggravated in the allograft group than the syngraft group, whereas Fer-1 administration remarkably decreased the collagen deposition in the allograft group. The results above demonstrated that Fer-1 alleviates the OB progression in orthotopic tracheal transplantation.

We then assessed the changes in GPX4 and PTGS2 and found decreased GPX4 and increased PTGS2 levels in the allograft group compared to the syngraft group. These changes were reversed by ferroptosis inhibitor Fer-1 in the allograft group (Fig. **2D** and **E**). Prussian Blue staining showed that the iron ion level in the allograft was obviously increased than the syngraft. However, Fer-1 treatment remarkably decreased the iron ion level in the allograft group (Fig. **2F**). TEM analysis showed that the mitochondrial volume was reduced, the membranes were more intense, the numbers of cristae were lessened, and the folded membranes were destroyed in the allograft group compared with those in the syngraft group. These severe mitochondria morphology impairments were restored by Fer-1 administration in the allograft group (Fig. **2G**).

The tissue iron and MDA levels were obviously increased in the allograft group more than in the syngraft group. However, this change was remarkably suppressed by Fer-1 treatment. The total GSH and SOD levels were significantly diminished in the allograft group compared with the syngraft group, and the Fer-1 treatment restored the GSH and SOD levels. Therefore, we concluded that ferroptosis contributes to OB progression after orthotopic tracheal transplantation (Fig. **2H**).

### 3.3 Iron Chelator Ameliorated OB and Ferroptosis After Orthotopic Tracheal Transplantation

**Fig. (3):**
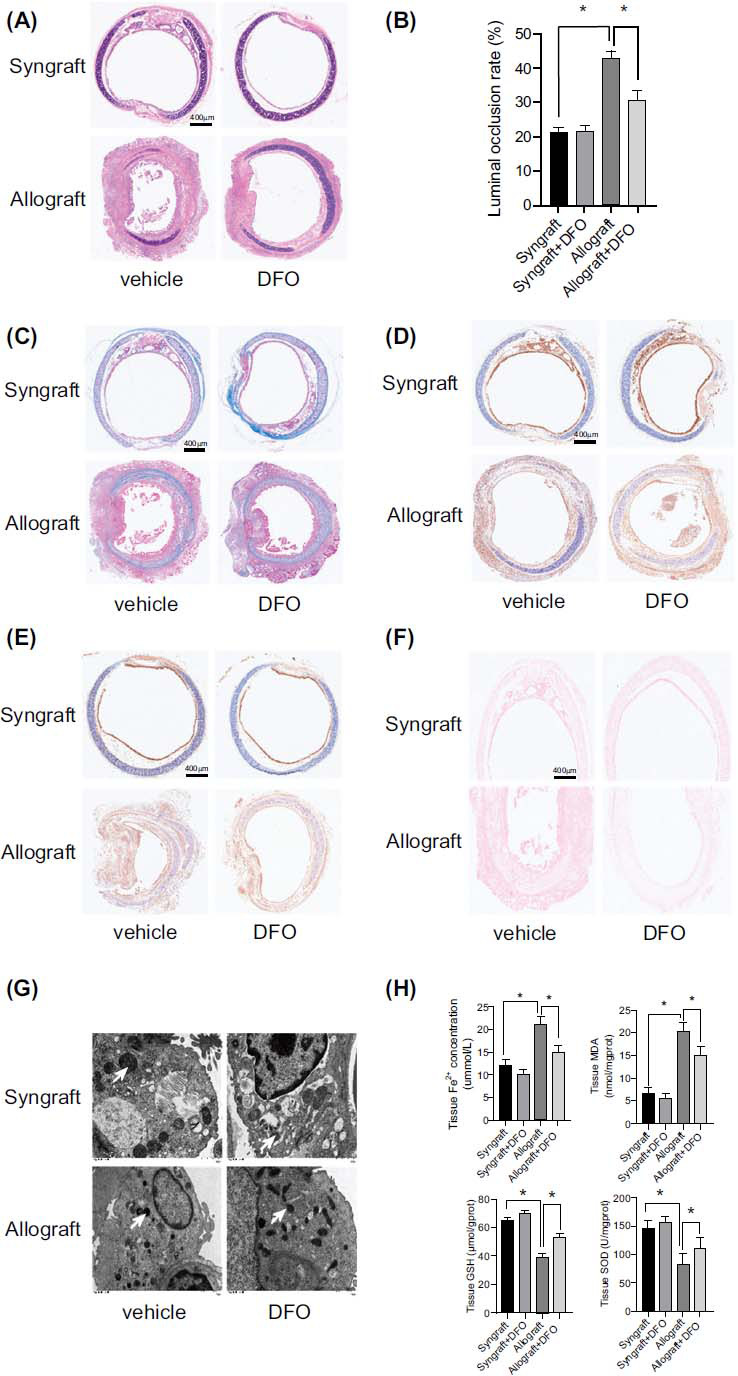
Iron chelator DFO ameliorated OB and ferroptosis after orthotopic tracheal transplantation: (**A** and **B**) The luminal occlusion rate was assessed in the HE-stained trachea slices. (**C**) Collagen deposition within the tracheal epithelium was evaluated by Masson’s trichrome staining. (**D** and **E**) GPX4 and PTGS2 expression was assessed by immunostaining. (**F**) Quantification of iron deposition in the graft trachea by Prussian blue staining. (**G**) TEM images of trachea tissue. The typical mitochondrial ultrastructure was shown as smaller mitochondria, condensed membrane, and fewer cristae in ferroptosis. ×6000 magnification, scale bar, 1 μm. White arrow, mitochondria. (**H**) Tissue iron concentration, MDA, GSH, and SOD levels were assessed. Data are expressed as mean± SEM. *P <0.05.

In order to investigate the role of iron in OB, an iron-chelated model induced with DFO was established. As shown in Fig. (**3A**-**C**), the luminal occlusion rates and tracheal epithelium fibrosis were significantly attenuated in the DFO-treated allograft compared to the vehicle-treated allograft. Immunostaining demonstrated increased GPX4 and decreased PTGS2 expression in the DFO-treated allograft compared to the vehicle-treated allograft group (Fig. **3D** and **E**).

As Prussian Blue staining shown in Fig. (**3F**), DFO treatment remarkably decreased the iron level in the allograft more than the vehicle-treated allograft. TEM analysis found that the mitochondrial membranes were less intense, and the number of cristae was increased in the DFO-treated allograft group than in the vehicle-treated allograft group (Fig. **3G**). The levels of iron and MDA content were obviously attenuated in the DFO-treated allograft compared to the vehicle-treated allograft group. Meanwhile, as shown in Fig. (**3H**), DFO restored the total GSH and SOD levels in the allograft group compared with the vehicle-treated allograft group. The results above illustrate that iron chelation alleviates ferroptosis and OB after orthotopic tracheal transplantation.

### 3.4 Iron Overload Exacerbated OB *via* Ferroptosis Activition

**Fig. (4):**
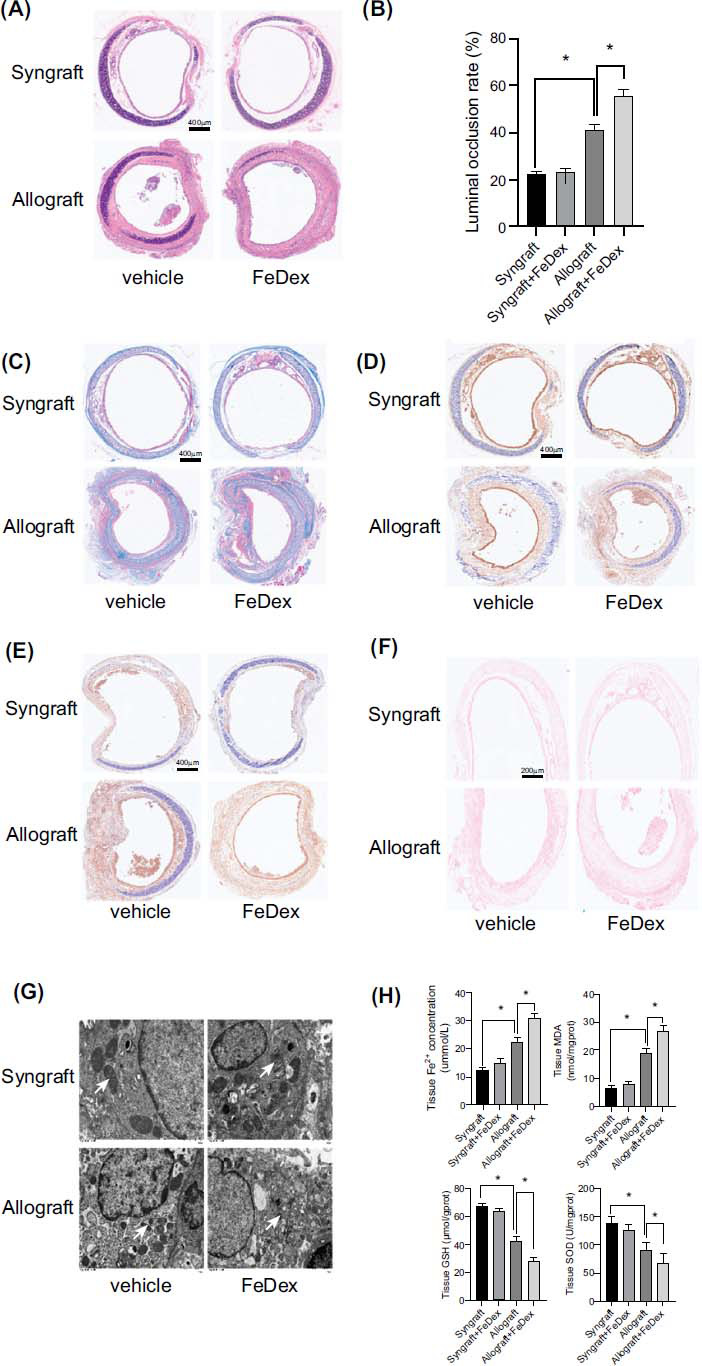
Iron overload-induced with FeDex exacerbated OB and ferroptosis after orthotopic tracheal transplantation: (**A** and **B**) The luminal occlusion rate was assessed in the HE stained trachea slices. (**C**) Collagen deposition within the tracheal epithelium was evaluated by Masson’s trichrome staining. (**D** and **E**) GPX4 and PTGS2 expression was assessed by immunostaining. (**F**) Quantification of iron deposition in the graft trachea by Prussian blue staining. (**G**) The typical mitochondrial ultrastructure was generally smaller, with a condensed membrane and fewer cristae. ×6000 magnification, scale bar, 1μm. White arrow, mitochondria. (**H**) Tissue iron concentration, MDA, GSH, and SOD levels were assessed. Data are expressed as mean± SEM. *P <0.05.

To explore the effects of iron overload on OB, iron dextran (FeDex) treated recipients were subjected to orthotopic tracheal transplantation. As shown in Fig. (**4A**-**C**), the luminal occlusion rates and tracheal epithelium fibrosis were significantly aggravated in the FeDex-treated allograft group compared to the vehicle-treated allograft group. Immunostaining demonstrated that decreased GPX4 and increased PTGS2 expression were observed in the FeDex-treated allograft compared to the vehicle-treated allograft (Fig. **4D** and **E**). As Prussian Blue staining is shown in Fig. (**4F**), FeDex treatment significantly increased the iron levels in the allograft group compared to those in thvehicle-treated allograft group. TEM analysis indicated that the mitochondrial membranes were more intense, and the number of cristae was lower in the FeDex-treated allograft than in the vehicle-treated allograft (Fig. **4G**).

The levels of iron and MDA content were obviously exacerbated in the FeDex-treated allograft compared to the vehicle-treated allograft. Meanwhile, FeDex decreased the GSH and SOD levels in the allograft group more than the vehicle-treated allograft group, as shown in Fig. (**4H**). Above all, these results illustrate that iron overload aggravates ferroptosis and OB after orthotopic tracheal transplantation.

**Fig. (5):**
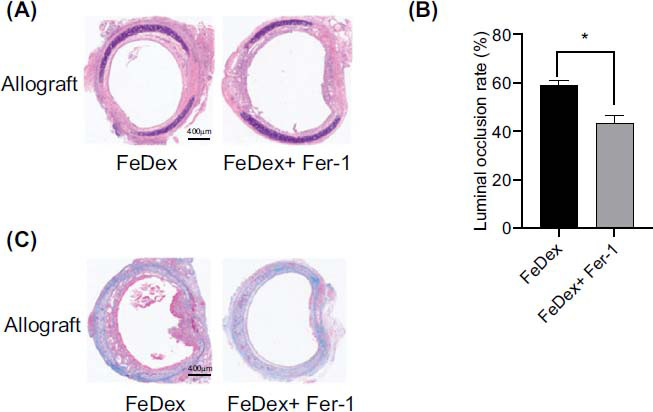
Inhibiting ferroptosis with Fer-1 prevented iron overload-induced OB. (**A** and **B**) The luminal occlusion rate was assessed in the HE-stained trachea slices. (**C**) Collagen deposition within the tracheal epithelium was evaluated by Masson’s trichrome staining. Data are expressed as mean± SEM. *P <0.05.

Furthermore, ferroptosis inhibitor Fer-1 intervention obviously prevented the luminal occlusion rates aggravation and collagen deposition in the FeDex-treated allograft group (Fig. **5**). These results demonstrated that iron overload aggravated OB after orthotopic tracheal transplantation *via* promoting ferroptosis.

## 4 DISCUSSION

The present findings of this research indicate that ferroptosis contributes to OB, and that ferroptosis inhibitor and iron chelator suppress ferroptosis to alleviate OB after orthotopic tracheal transplantation. Our results demonstrated that iron overload-induced ferroptosis is involved in OB, which may be a potential therapeutic target for OB prevention.

OB is a complicated pulmonary syndrome that causes tiny airway inflammation and fibrosis, which progressively obstructs airflow and results in end-stage respiratory failure. It is an intricate pathological process with multiple cell types, mediators, and signaling pathways, which is always the typical complication of lung transplantation, hematopoietic stem cell transplantation, or exposure to toxic fumes [38-41]. The pathogenesis of OB is not fully understood until now, but previous investigations suggested that various types of programmed regulated cell death play a vital role in tissue damage and remodeling of the OB [42]. Apoptosis, necroptosis, pyroptosis, and ferroptosis are four distinct forms of programmed regulated cell death that differ in their molecular mechanisms, morphological features, and immunological consequences. The relationship between apoptosis, necroptosis, pyroptosis, and OB is complex, context-dependent, and not fully clarified, but some possible mechanisms have been proposed [43-46]. Apoptosis is a physiological process that eliminates unwanted or damaged cells in a controlled programmed procedure, which is a common complication that affects the graft success rate [47, 48]. Epithelial cell apoptosis may be triggered in various conditions, such as ischemia-reperfusion injury, acute rejection reaction, infection, or chronic allograft rejection [49, 50]. Alho *et al*. ever revealed that apoptosis level increased along with epithelial injury, which proved that epithelial apoptosis was an initiating factor to the OB [43]. This process can also be impaired by various conditions, such as viral infections, oxidative stress or immunosuppressive drugs, resulting in persistent inflammation, tissue injury and fibrosis [51-53]. Epithelial cell, endothelial cell, and lymphocyte apoptosis can consequently induce epithelial damage, loss of bronchiolar integrity, and the fibroblasts activation, leading to a cascade of events, such as cytokine release, extracellular matrix deposition, and smooth muscle proliferation, which finally result in airway remodeling and obstruction [37, 54, 55]. Necroptosis is a programmed cell death mode that involves the Receptor-Interacting Protein Kinase 3 (RIPK3) and Mixed Lineage Kinase domain-like protein (MLKL) activation, which cause plasma membrane rupture and excessive inflammation [56, 57]. Necroptosis can release DAMPs that induce the adaptive immune cells and trigger alloimmune and rejection responses additionally [58]. A previous study revealed that necrotic cell aggravates ischemia reperfusion injury and lead to poor prognosis of lung transplantation [59, 60]. However, there is a lack of research on the potential role of necroptosis on OB.

Pyroptosis is another caspase-independent programmed cell death that is caused by the inflammasomes, which are immunogenic and pro-inflammatory since they promote inflammatory factors release [61, 62]. Pyroptosis leads to the cleavage of gasdermin D (GSDMD), which forms pores in the plasma membrane and induces inflammatory cytokines and other cellular contents, such as interleukin-1β (IL-1β) and IL-18 [63]. Pyroptosis can prompt the activation and recruitment of neutrophils and macrophages in the airways of OB. These cells can further produce excess amount of ROS, proteases, and cytokines that cause tissue damage, remolding, and fibrosis [64, 65]. Yan *et al*. demonstrated that pyroptosis can directly affect the epithelial cells lining the airways, causing the barrier function injury and expose the underlying tissue to injury and infection [66]. Pyroptosis may modulate the allograft immune response and the balance between fibrotic and anti-fibrotic processes in the lung, which results in terminal airway fibrosis [67]. Moreover, these forms of programmed cell death can interact with each other and modulate their physiological effects. For instance, apoptosis may inhibit necroptosis and pyroptosis by cleaving and inactivating RIPK3 and GSDMD, whereas necroptosis and pyroptosis induce apoptosis by activating caspase-8 and caspase-3, respectively [68, 69].

In terms of iron deposition, they are at high risk of tissue injury and fibrosis. Baz *et al*. found that iron concentration in the bronchoalveolar lavage fluid, alveolar macrophages, and bronchiolar epithelium of the lung transplantation recipients were much higher as compared with normal volunteers [70]. Chu *et al*. ever reported a 61-year old patient suffered from necrosis, stenosis, and near-complete obstruction of distal right bronchus intermedius due to exposure to iron aspiration for 4 weeks [71]. Another case of interstitial lung disease and OB induced by iron dust exposure was reported by Yi *et al*. [7]. When iron is deposited in bronchial epithelial, it is converted into a toxic ferric form, which causes mucosal injury, acute inflammation, and fibrosis [72, 73]. Reid *et al*. proposed that lung allograft could be damaged by iron-induced oxidative stress in OB, which NO and ROS may further aggravate. Prussian blue and immunohistochemical staining of lung allograft showed an iron deposition in the alveolar, interstitium and epithelium. Abnormally high iron ion and homeostatic proteins were observed in the lung allografts, and their levels tend to increase over time [74, 75]. This proves an unphysiological iron metabolism and a critical impact of catalyzed oxidative stress after lung transplantation.

In the present research, we first explored the role of ferroptosis in OB development after orthotopic tracheal transplantation in mice. It has not been reported yet now. We found that ferroptosis and FeDex-induced iron overload contribute to OB and that ferroptosis inhibitor Fer-1 and iron chelator DFO suppress ferroptosis and alleviates OB. Iron overload significantly exacerbated the luminal occlusion rate and enhanced fibrosis in the subepithelial layer of the graft trachea. Iron overload increased the expression of ferroptosis markers, such as lipid peroxidation MDA and PTGS2, in the transplanted trachea. In order to verify the impact of ferroptosis in iron overloadinduced OB, a specific ferroptosis inhibitor (Fer-1) was applied in our study. The results demonstrated that Fer-1 significantly reversed the iron overloadinduced luminal occlusion rate and fibrosis in the graft trachea.

However, several limitations still need to be figured out in the next step. First, the orthotopic tracheal transplantation model in mice was applied in our study, which may not fully reflect the clinical situation of human lung transplantation since there is always a potential species-specific difference. Further validation in human samples and the safety and efficacy of ferroptosis inhibitors in clinical settings should not be neglected. Second, the underlying molecular mechanism and signaling pathway involved in iron overload induced ferroptosis and OB remains to be revealed. Furthermore, the potential interactions between iron-induced ferroptosis and other cell death, such as pyroptosis and necroptosis, could also be explored.

## CONCLUSION

**Fig. (6):**
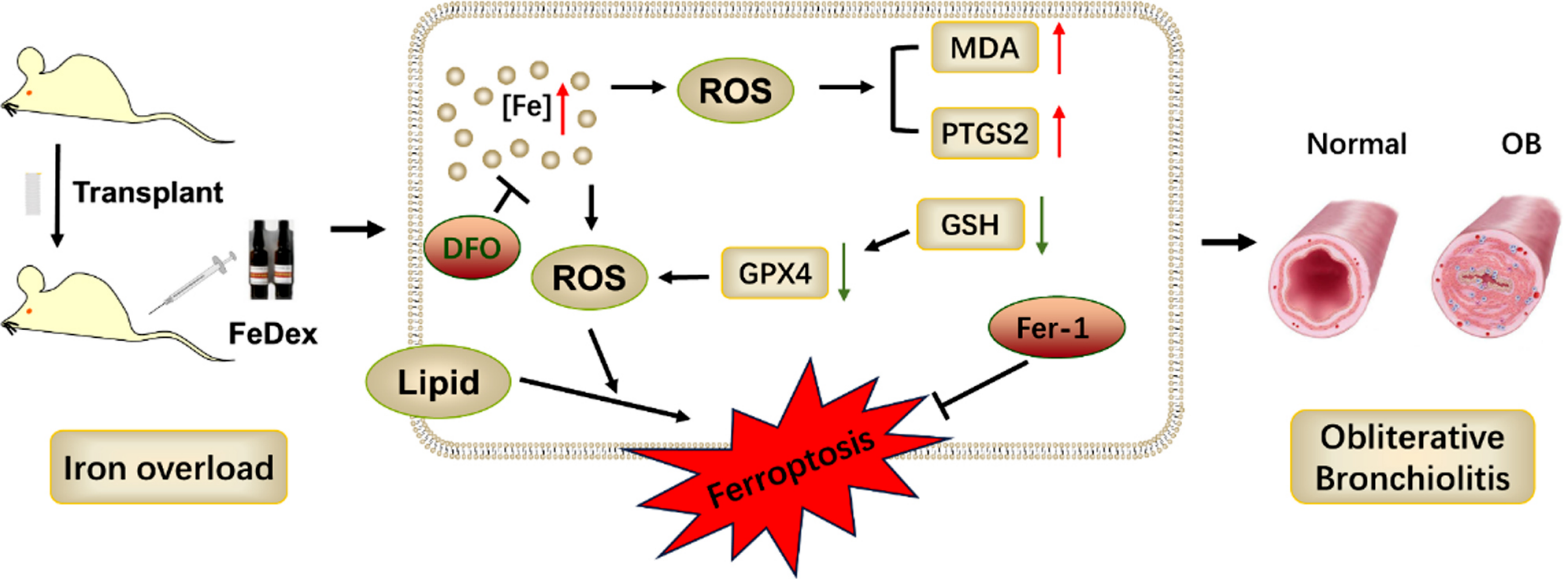
The schematic figure for the iron overload-induced ferroptosis involving in OB.

In conclusion, the present study demonstrated that iron dextran overload exacerbates OB while inhibiting ferroptosis *via* Fer-1 and DFO, which shows a therapeutic potential role for OB progression (Fig. **6**). Therefore, we speculate that targeting iron overload or ferroptosis could be a novel therapeutic approach for preventing OB after lung transplantation. Further experiment is needed to illustrate the molecular mechanisms of ferroptosis in the pathophysiology process of OB after lung transplantation.

## Data Availability

The data and supportive information are available within the article.

## LIST OF ABBREVIATIONS

OB: Obliterative Bronchiolitis
ROS: Reactive Oxygen Species
CLAD: Chronic Lung Allograft Dysfunction
Fer-1: Ferrostatin-1
GSH: Glutathione
PTGS2: Prostaglandin-endoperoxide Synthase 2
GPX4: Glutathione Peroxidase 4
BCA: Bicinchoninic Acid
SOD: Superoxide Dismutase
MDA: Malondialdehyde
IL-1β: Interleukin-1β
GSDMD: Gasdermin D
RIPK3: Receptor-Interacting Protein Kinase 3
MLKL: Mixed Lineage Kinase domain-like protein
